# Drugs for Pain Management in Shock Wave Lithotripsy

**DOI:** 10.1155/2011/259426

**Published:** 2011-11-03

**Authors:** Christian Bach, Faruquz Zaman, Stefanos Kachrilas, Priyadarshi Kumar, Noor Buchholz, Junaid Masood

**Affiliations:** Department of Urology, Barts and The London NHS Trust, West Smithfield, London EC1A 7BE, UK

## Abstract

*Objective*. With this review, we provide a comprehensive overview of the main aspects and currently used drugs for analgesia in shockwave lithotripsy. *Evidence Acquisition*. We reviewed current literature, concentrating on newer articles and high-quality reviews in international journals. *Results*. No standardized protocols for pain control in SWL exist, although it is crucial for treatment outcome. General and spinal anaesthesia show excellent pain control but are only recommended for selected cases. The newer opioids and nonsteroidal anti-inflammatory drugs are able to deliver good analgesia. Interest in inhalation anaesthesia with nitrous oxide, local anaesthesia with deep infiltration of the tissue, and dermal anaesthesia with EMLA or DMSO has recently rekindled, showing good results in terms of pain control and a favourable side effect profile. Tamsulosin and paracetamol are further well-known drugs being currently investigated. *Conclusion*. Apart from classically used drugs like opioids and NSARs, medicaments like nitrous oxide, paracetamol, DMSA, or refined administration techniques for infiltration anaesthesia show a good effectiveness in pain control for SWL.

## 1. Introduction

With the first serial lithotripter, the Dornier HM3 (Human Model 3), introduced in 1983 by Chaussy et al., treatment was painful and therefore the procedure had to be performed under general or spinal anaesthesia [1]. 

The subsequent generations of shock wave lithotripsy (SWL) machines were optimized towards patient comfort and treatments became less painful. Modification of the shock wave generators towards a wider aperture led to a reduced intensity of the shock waves on skin entry level, a smaller focal zone and a lower energy output made the treatment under analgesia rather than under anaesthesia possible [1].

But, also with a second-generation machine, the Dornier HM4, still 95% of SWL patients experienced pain during the treatment [2]. Especially the piezoelectric lithotripters are an example for a very wide area of entry over the skin with a very small focal point. Treatment with these machines was hoped to become pain-free [3]; nevertheless, even with the modified and adapted third generation machines, nearly 30% of all patients still reported severe pain when treated without analgesia [3]. 

Clinical outcomes and success as measured in terms of stone-free rate after SWL is strongly correlated to pain experienced during treatment [4]. Pain during SWL treatment may lead to defocussing through voluntary or involuntary patient movement and can cause increased respiratory motion, both resulting in a reduced hit rate with a reduced stone fragmentation and a lower overall stone clearance. Additionally, pain which reduces the patient's compliance, can limit the shockwave energy and number and may lead to more complications like a higher rate of kidney haematomas due to a rise in blood pressure. It is evident that despite latest generation machines, SWL is still a painful procedure and adequate analgesia is mandatory in order to achieve an optimal result and patient compliance [5].

The optimal anaesthesia technique should be easy to administer and have a high efficacy and minimal side effects. Adequate analgesia, sufficient sedation, and rapid recovery are needed, the latter especially in an outpatient setting. To achieve optimal pain control, the patient needs to be closely monitored regarding this during the treatment, ideally by the operator who is controlling the applied energy level and can immediately reduce it, should pain occur. For protocols, where opioids or sedatives are administered, a second person other than the operator needs to be present to monitor the patient's vital functions and to deliver drugs according to the patient's needs [6, 7]. 

To date, there are no guidelines for pain therapy during SWL treatment and a variety of treatment protocols and drugs are being used. Traditionally, nonsteroidal, anti-inflammatory drugs (NSAIDs) such as diclofenac, ketorolac and piroxicam, as well as a variety of opioids like morphine, pethidine, and fentanyl are employed sometimes in combination with sedative hypnotics. 

In this review, we emphasize newer drugs and drugs in which interest has been rekindled recently such as inhalation anaesthesia with nitrous oxide, local infiltration techniques, topical creams, newer opioids, and NSAIDs.

## 2. Pathogenesis of Pain during SWL

Shock-wave-induced pain is usually described as stinging and sharp. Its pathogenesis is not yet totally understood but cavitation seems to play a key role, rather than direct mechanical effects on nociceptive nerve endings [8, 9]. Formation, movement, and implosion of the shock wave generated microbubbles in body fluids or tissues lead to stimulation of the superficial nociceptors in the skin as well as the deeper, visceral nociceptors in the renal capsule, periost, pleura, peritoneum, and muscles [9, 10].

A second component of shock-wave-related pain is the movement of the stone caused by the impact of the shock wave [11].

Several physical variables influencing treatment-related pain have been identified: an important role is played by the type of the shockwave source, size, and site of stone burden (e.g., upper-pole stone near the ribs), peak pressure of the shockwaves, diameter of the focal zone, and size of the aperture of the shockwave source reflecting the area of shockwave entry at the skin [12–14].

Furthermore responsible for pain perception during SWL are patient-related factors like age, gender, and body habitus [4], and young female patients, anxious and depressed patients or thin patients (shockwaves more concentrated) all experience more pain during SWL [15, 16].

## 3. Contemporary Drugs: Characteristics and Side Effects

### 3.1. General Anaesthesia (GA)

General anaesthesia (GA) for SWL treatment offers optimized pain control and controlled respiratory excursion. This creates optimal conditions for stone targeting and consecutive fragmentation. In modern anaesthetic practice, GA is considered to be safe with a low morbidity. However, it seems preferable to avoid, if not mandatory, especially in high-risk patients. Disadvantages are the need to involve an anaesthetic specialist and the need for postoperative recovery, increasing the overall costs and making GA less suitable in the common outpatient SWL setup of most urological departments. Solely in children or in extremely anxious patients, it is still the preferred option. 

Other potential indications are particularly long treatments as in patients with bilateral stones, concomitant renal and ureteral stones or in patients with very hard calculi (cystine, calcium oxalate monohydrate, or brushite), which are known to be resistant to fragmentation and require high, potentially painful energy levels [14, 17].

### 3.2. Inhalation Anaesthesia with Nitrous Oxide

Nitrous oxide is a medical anesthetic gas and on the market as Entonox, a mixture of 50% nitrous oxide and 50% oxygen. It was discovered in 1776 by Joseph Priestly [18] and constitutes another analgesic option for SWL treatment. 

This colourless gas is highly soluble in blood and the arterial concentration reaches a plateau 10 minutes after commencing the inhalation. It diffuses rapidly through the cellular membranes, does no bind to haemoglobin, and is eliminated unchanged via the lungs. The analgesic effect commences 20 to 30 seconds after inhalation and a peak effect is reached after 3 to 5 minutes. Reflexes of coughing and airway protection are not noticeable altered [18]. This rapid onset and quick loss of effect make nitrous oxide an attractive option for day-case procedures. Especially in treatments of short duration such as SWL, Entonox can be used in spontaneously breathing patients to provide analgesia. Regarding its analgesic effect, the concentration of 30% of nitrous oxide is reported to be equivalent to 10–15 mg of morphine [19].

Adverse effects of Entonox are mainly transient nausea and light headedness. A further issue can be slight cardiac depression; therefore, the gas should be used carefully in patients with congestive heart failure and in those with obstructive airway disease [20]. It is contraindicated in patients with pathological air-filled body cavities (pneumothorax or obstructed bowels) as nitrous oxide is diffusing into these, consecutively increasing volume and pressure therein. 

The use of Entonox during SWL was reported in only one RCT study up to now; 150 patients undergoing treatment where randomized into 3 groups, one to receive Entonox, the other to have intravenous pethidine, and the last one to inhale compressed air. A significantly reduced procedure-related pain (*P* = 0.001) for nitrous oxide could be shown and it proved as effective as intravenous administered pethidine [21].

### 3.3. Spinal Anaesthesia

Spinal anaesthesia with subarachnoidally injected local anaesthetics or opioids has shown excellent results in terms of analgesia which makes it suitable for a variety of surgical procedures. Its use for SWL with application of lidocaine and sufentanil has been repeatedly described and again the results in terms of analgesic effect are excellent [22–25]. 

Sufentanil is considered to be the safer alternative to lidocaine as it preserves motor and sensory function, facilitating earlier ambulation and discharge [14, 24]. An undesirable side effect of sufentanil is pruritus, and inconvenient for the urologist is the need for active patient monitoring [22, 25] and due to the risk of respiratory depression it should be used with caution, especially in the elderly or in patients with obstructive airway disease [3]. Furthermore, spinal anaesthesia it is a time-consuming procedure; an anaesthetist must be involved and patients have a prolonged recovery time due to residual sympathetic blockade.

### 3.4. Infiltrating Local Anaesthesia

The analgesic efficacy of subcutaneous (SC) infiltration of lidocaine with 1 : 100.000 epinephrine for SWL has early been demonstrated. However, infiltrating a relatively large area (200 cm^2^) with significant discomfort at the site of injection and the need for additional IV analgesia and sedation made this approach inconvenient [3, 26].

Prilocaine has an equal analgesic efficacy, a more rapid onset, a similar duration, and is due to its rapid metabolism, less toxic. The target area is usually infiltrated only 1-2 min before the treatment. Yilmaz et al. infiltrated a limited area of 30 cm^2^ representing the entry area of the shockwave on the skin with prilocaine. Compared to intra-muscularly administered diclofenac, less supplementary analgesia (fentanyl) was needed [27]. By infiltrating 20 mL prilocaine subcutaneously and deep lumbar, an even better analgesic effect could be shown, thus minimizing the need for additional analgesia [28]. Recently, the combination of subcutaneous infiltration of 10 mL 2% lidocaine and 10 mL 0.5% bupivacaine, administered 5 min before the treatment has shown to be sufficiently analgesic during SWL treatment and only rarely additional intravenous analgesia was needed [29].

### 3.5. Dermal Anaesthesia

EMLA (Eutectic Mixture of Local Anaesthetics) cream is an eutectic mixture of lidocaine 2.5% and prilocaine 2.5% for topical application. It is commonly used for dermal anaesthesia, for example, in blood sampling, venous catheterization, condylomata acuminata excision, or debridement of leg ulcers. Its penetration depth through intact skin is about 4 mm after 60 minutes [30, 31]. Its use in SWL for pain relief has early been reported [32]. 

To maximize its local analgesic effect, EMLA should be applied under an occlusive dressing. Due to its comparable weak analgesic potential, it is usually used in SWL together with additional analgesic agents like opioids or NSAIDs [13]. The ultrasound-jelly-like viscosity is a favourable characteristic since it acts as a coupling medium [17]. A further advantage of this form of analgesia is its simplicity and noninvasiveness, avoiding the side effects of IM or IV analgesic agents. To achieve a maximal analgesic effect, the cream needs to be applied 60 to 90 minutes before the procedure, application is not particularly time consuming but a good timing is crucial [33, 34].

Whilst earlier studies showed no reduction in the need for additional opioid analgesics after application of EMLA cream [35], a more recent randomized study comparing oral Diclofenac, EMLA cream, and a combination of both reported a favourable outcome. There was no need for additional parenteral analgesics in the latter group, and as a result a better overall stone-free rate and reduced procedure related complications could be shown [36].

Dimethyl sulfoxide (DMSO) acts as a topical analgesic and is at the same time a vehicle for the transport of other local acting pharmaceuticals through the skin [36]. In addition, it has anti-inflammatory, muscle relaxing, hydroxyl radical scavenger, and diuretic effects [37]. In combination with lidocaine, it has shown to deliver a better pain control compared to EMLA cream [38].

### 3.6. Opioids

Frequently used drugs are pethidine, the first synthetic opioid, fentanyl, tramadol, alfentanil, remifentanil, and sufentanil and a variety of administration techniques such as intramuscular bolus (IM) injection, or intravenous (IV) on-demand patient-controlled analgesia [11, 36].

Opioids are delivered either alone or in combination with other forms of analgesia or sedation [39–41]. Due to side effects like nausea, vomiting, and respiratory depression, opioids require ECG, blood pressure and oxygen saturation monitoring which limits their use, especially in an outpatient setting [42, 43]. The combination of opioids and benzodiazepines has shown to decrease the need for IV opioid analgesia and led to high patient satisfaction [44].

Fentanyl is a potent synthetic narcotic, has a rapid onset and a short duration of action. It is a strong agonist at the *μ*-opioid receptors, provides an acceptable analgesia condition during SWL, and is therefore commonly used. One of its drawbacks is the need for continuous noninvasive pulse oximetry due to its marked respiratory depressive effect [45]. The combination of fentanyl and propofol has shown to be even more potent analgesic in SWL at the costs of more pronounced side effects like respiratory depression, decreasing oxygen saturation, nausea, vomiting, drowsiness, and hypersensitivity reactions [40, 46]. Remifentanil and sufentanil are more recent developments and have been used in SWL alone or in combination with sedatives, local anaesthetics, or other analgesics [42, 47–49]. They are equal in their analgesic potency and have shown to lead to similar patient's and surgeon's satisfaction. Remifentanil has the more convenient side effect profile leading to lesser respiratory depression, nausea, and vomiting [42]. Both of them can safely be applied in patients with hepatic or renal diseases [49]. 

Tramadol hydrochloride is a relatively weak, central active opioid analgesic, acting as an agonist at the *μ*-opioid receptors, inhibiting the reuptake of noradrenalin and releasing 5-hydroxytryptamine (Serotonin) [50]. Respiratory depression is not clinically significant in normal doses [51]. For a dose of 100 mg of tramadol IV, efficient pain control during SWL has been reported, despite relatively higher rate of side effects, especially a high incidence (25%) of nausea and vomiting [49, 52]. The mechanism triggering nausea and vomiting remains unclear but seems to be related to its central effect on opioid receptors [45].

Patient-controlled analgesia avoids problems of IV opioid overadministration such as respiratory depression and the need for intra- and postoperative patient monitoring [53]. Every time the patient presses a button, a small predefined dose is delivered and a safety mechanism does not release another dose before a set time. Theoretically, this option provides more pain control and more effective targeting should be possible [54]. Nevertheless it requires an active intelligent patient, the device is expensive and problems of malfunctioning can occur. Furthermore, patients need to be actively monitored and continuous noninvasive pulse oximetry is mandatory during and up to 2 hours after administration [11, 45].

Staff administering the above listed substances should be trained in cardiopulmonary resuscitation and the appropriate reversal agents should be at hand: naloxone for opioid reversal and flumazenil for benzodiazepine reversal [17].

### 3.7. Nonsteroidal Antiinflammatory Drugs (NSAIDs)

Diclofenac, ketorolac, and piroxicam are widely used for SWL as they have a good analgesic effect [4, 17, 36]. By inhibiting the enzyme cyclooxygenase (COX), NSAIDs reduce the synthesis of prostaglandins, which act as messengers in inflammatory processes; they reduce the renal blood flow, the renin release, and the glomerular filtration rate. On the other hand, by blocking the synthesis of thromboxane, they also have an antithrombotic effect and affect haemostasis, potentially leading to a prolonged bleeding time [4]. To distinguish them from steroids, which have (amongst many others) a similar prostaglandin depressing, antiinflammatory action, the term “nonsteroidal” is used. In contrast to many other analgesic drugs, NSAIDs are nonnarcotic. 

They can be given orally, intravenously, intramuscularly, or rectally [11, 27, 36]. They are not indicated in patients with a history of intestinal ulcers, renal and hepatic insufficiency, chronic obstructive pulmonary disease (COPD), and coagulation disorders allergies [14]. Depending on the way of administration, they should be given 30 to 60 minutes before the procedure [36]. Side effects include gastrointestinal disturbances, hypersensitivity reactions, and coagulation disorders due to cyclooxygenase inhibition [55].

Lornoxicam is a short-acting, nonsteroidal anti-inflammatory agent, inhibiting COX-1 and COX-2 and belonging to the oxicam group [56]. A single dose of 8 mg lornoxicam has proved to be effective for pain relief during SWL, being superior 20 mg of tenoxicam, which is another drug of the same class [44, 57].

Selective cyclooxygenase-2-inhibitors (COX-2) are particularly interesting for pain relief during or after surgical procedures such as SWL, as they have no effect on thromboxane and a potential better side effect profile. They are not yet widely in use for pain relief during SWL, only parecoxib, which can be administered IV or IM has recently been under investigation, showing a rather limited analgesic effect and proving to be remarkably less effective than fentanyl [58]. 

Rofecoxib is another COX-2 inhibitor, which showed to provide good pain relief *after* SWL treatment, but in the meantime this substance has been withdrawn from the market due to safety concerns [59].

### 3.8. Paracetamol

Paracetamol is a derivative of *p*-aminophenol. It is not counted to the NSAIDs because it has no anti-inflammatory effect. It is commonly used for pain control after surgical procedures.

There is only one study in the literature proving the efficacy of paracetamol in SWL [60].

### 3.9. Tamsulosin 

Tamsulosin is an *α*_1a_-selective alpha blocker and widely used in urology for the symptomatic treatment of urinary outflow obstruction due to benign prostate enlargement. It has also been shown to increase stone-free rate after SWL treatment and to reduce the need for opioid analgesics in patients with renal colics [61]. It was currently under investigation for pain relief during SWL treatment but up to now, no significant benefit could be shown [62].

### 3.10. Multimodal Analgesia

Multimodal (or balanced) analgesia represents a modern approach to pain control during surgery by administering a combination of opioid and nonopioid analgesics like local anesthetics, NSAIDs, COX-2 inhibitors, acetaminophen, ketamine, dextromethorphan, *α*-2 agonists, gabapentin, magnesium, and neostigmine [63]. These nonopioid analgesics are increasingly being used as adjuvants before, during, and after surgery to reduce the amount of administered opioids and to facilitate thereby the recovery process. Such an approach is already common practice for pain control during SWL treatment, where in many cases a nonopioid like EMLA [13], subcutaneous local anaesthetic [27] or NSAIDs [56] is administered beforehand and supplementary analgesic needs are covered with intravenous administered opioids.

## 4. Summary

Even with latest generation lithotripters, SWL is still a potential painful procedure and sufficient analgesia is mandatory for good treatment results.

General anaesthesia should be reserved for selected cases and for the treatment of children; the same applies to spinal anaesthesia. Both guaranty optimal pain control but have high demands of manpower, resources, and a lead to prolonged recovery, making them less suitable for SWL as an outpatient procedure. 

In this context, inhalational anaesthesia with nitrous oxide is another, very interesting option, as it delivers good analgesia, is easy to administer, and does not lead to prolonged recovery. 

Subcutaneous infiltration with local anaesthetics has proven to be effective in terms of pain control and safe, as it avoids side effects of opioids.

The concept of dermal anaesthesia is not new but remains an interesting option due to the simplicity of its use and the convenience for the patient. Especially the combination of lidocaine with DMSO lately showed promising results.

Opioids, sometimes in combination with sedatives are the classical substances for pain control in SWL. They have a very good analgesic action, but also inconvenient side effects, require patient monitoring and lead to a delayed patient discharge. Sedoanalgesia and patient-controlled analgesia lead to good pain relief and patient satisfaction but are costly and likewise limited in an outpatient setting. 

NSAIDs are very convenient for both surgeon and patient. They are easy to administer, do not require patient monitoring and patients can be discharged immediately after the procedure. The newer substance lornoxicam has proven to have a good analgesic effect, whereas selective cyclooxygenase-2-inhibitors have not yet proven to be equally effective.

Substances like Paracetamol and Tamsulosin are widely in use for other indications and have recently been in the focus. Their very convenient side effect profile makes them very interesting, though the analgesic component, especially of Tamsulosin, is not convincing.

Despite its crucial importance for the outcome, no standardized protocols for pain control in SWL exist. Whatever substances or ways of administration are used, it remains the duty of the operator to ensure a pain-free treatment, for example, by delivering additional opioids on demand.
